# Reviewed article: Pinto ABD, Thomás BVS, Lima LMR, Maia JCS, Sousa AFM, Nepomuceno DB. Epidemiologic aspects and spatial distribution of human visceral leishmaniasis: a cross-sectional study, Crateús, 2007-2023. Epidemiol Serv Saude. 2025:34;e20250155

**DOI:** 10.1590/S2237-96222025v34e20250155.a

**Published:** 2025-09-08

**Authors:** Patricia Matias Pinheiro

**Affiliations:** 1 Empresa Brasileira de Serviços Hospitalares, Brasília, DF, Brazil Brasília DF Brazil

## Peer review A

## Questionnaire

Does the manuscript contain new and significant information to justify publication? Yes

Does the Abstract (Summary) clearly and accurately describe the content of the article? Yes

Is the problem significant and concisely stated? Yes

Are the methods described comprehensively? Yes

Are the interpretations and conclusions justified by the results? Yes

Is adequate reference made to other work in the field? Yes

## Manuscript Structure

Length of article is: Adequate

Number of tables is: Adequate

Number of figures is: Adequate

Please state any conflict(s) of interest that you have in relation to the review of this paper: None

Interest: Avarage

Quality: Good

Originality: Avarage

Overall: Avarage

Recommendation: Minor revision

## Comments

O delineamento escolhido, segundo os autores foi: transversal, qualitativo e quantitativo, retrospectivo e descritivo.

Na minha opinião, não há uma análise qualitativa. Para os dados do IBGE necessita informar de qual censo foram obtidos os dados.

Os resultados estão apresentados de forma conclusiva, no entanto, tem interesse puramente local.

A discursão dos resultados mostra os principais achados, fazendo paralelos com contexto de municípios próximos. Há discussão de limitações, contudo pouco se discute como minimizá-las.

